# Transcription factor CCAAT/enhancer binding protein alpha up-regulates microRNA let-7a-1 in lung cancer cells by direct binding

**DOI:** 10.1186/s12935-016-0294-5

**Published:** 2016-03-09

**Authors:** Yani Lin, Jian Zhao, Xiaoyan Hu, Lina Wang, Liming Liang, Weiwen Chen

**Affiliations:** Department of Biochemistry and Molecular Biology, School of Medicine, Shandong University, Jinan, 250012 People’s Republic of China; Department of Thoracic Surgery, Qilu Hospital, Shandong University, No. 107 Wenhuaxi Road, Jinan, 250012 Shandong People’s Republic of China

**Keywords:** C/EBPα, let-7a-1, Promoter, Lung cancer

## Abstract

**Aims:**

The transcription factor CCAAT/enhancer binding protein α (C/EBPα) and microRNA (miRNA) let-7a-1 act as tumor suppressors in many types of cancers including lung cancer. In the present study, we aim to investigate whether let-7a-1 is a novel important target of C/EBPα in lung cancer cells.

**Methods:**

The DNA sequence of the 2.1 kb let-7a-1 promoter was analyzed with MatInspector 4.1 (http://www.genomatix.de). Human lung cancer cell lines A549 and H1299, and human cervical cancer cell line Hela were used for transfection. Total RNA was extracted from cells using Trizol reagent and pri-let-7a-1 mRNA expression was measured using quantitative real-time polymerase chain reaction. Western blotting was performed to detect C/EBPα protein expression. To test whether C/EBP-α could up-regulate the expression level of let-7a at transcription level, dual-luciferase reporter gene assay was carried out. To determine whether C/EBPα could bind let-7a-1 promoter, electrophoretic mobility shift assay was employed. To further confirm the direct targeting let-7a-1 promoter by C/EBPα, chromatin immunoprecipitation was used.

**Results:**

Both C/EBPα and let-7a-1 were down-regulated in lung cancer A549 and H1299 cells, but up-regulated in Hela cells. Transfection and reporter gene assay showed that C/EBPα increased the expression of let-7a-1 at transcription level. Bioinformatics assay identified four putative C/EBP elements within let-7a-1 promoter. Dual-luciferase reporter gene, electrophoretic mobility shift assay and chromatin immunoprecipitation assays demonstrated that these four elements mediated the up-regulation effect of C/EBPα on let-7a-1.

**Conclusions:**

The present study reveals that decreased C/EBPα contributes to the down-regulation of miRNA let-7a-1 in lung cancer cells.

## Background

MicroRNA (miRNA) let-7 family plays pivotal roles in regulating carcinogenesis [1]. The let-7 family functions as tumor suppressors in multiple tumor types. They can inhibit the expressions of multiple oncogenes, including rat sarcoma viral oncogene homolog (RAS), high mobility group AT-hook 2 (HMGA2) and v-myc avian myelocytomatosis viral oncogene homolog (MYC) [2–4]. Down-regulation of let-7 family members is observed in multiple carcinomas, especially in lung cancer [5–7]. Therefore, the mechanism of down-regulation of let-7 family members in various cancers needs to be elucidated, and will help understand the regulation mechanism of let-7 in carcinogenesis.

Humans have 13 let-7 family members [8], including let-7a-1, let-7a-2, let-7a-3, let-7b, let-7c, let-7d, let-7e, let-7f-1, let-7f-2, let-7g, let-7i, miR-98, and miR-202, which are located in nine different loci on chromosomes 3, 9-12, 19, 21, 22, and X. A total of ten mature let-7-family sequences are produced from the 13 precursor sequences. For example, pre-let-7a-1, pre-let-7a-2 and pre-let-7a-3 produce mature let-7a, while pre-let-7f-1 and pre-let-7a-2 produce mature let-7f. At transcriptional level, it has been reported that let-7 family members can be inhibited by oncogenic transcription factor MYC in lymphoma and hepatocellular carcinoma cells [9, 10]. It is also shown that transcriptional repressor jumonji AT-rich interactive domain 1B (JARID1B) protein binds to let-7e promoter, leading to the repression of let-7e and the promotion of breast tumor cell cycle [11]. In addition, nuclear factor-kappa B (NF-κB) can enhance the transcription of let-7a-3/let-7b cluster in Hela cells [12]. At posttranscriptional level, the main regulation factor for let-7 family members is lin28, which blocks the maturation of pri-let-7 by binding the loop region of the pri-let-7 stem-loop [13, 14]. However, the understanding about the regulation of let-7 is still limited. Three let-7 family members, let-7a-1, let-7f-1 and let-7d-1, form an intergenic miRNA cluster on chromosome 9p22.32, which is encoded by a single polycistronic transcript driven by a single promoter. The three members are the most abundant species of let-7 family, together accounting for about 24 % of all let-7 precursors [5, 15]. In addition, let-7a-1 is reported to be down-regulated in lung cancer, suppressing the proliferation of lung cancer cells by its overexpression [3, 5].

The CCAAT/enhancer binding protein α (C/EBPα) is a differentiation-inducing transcription factor that belongs to a family of basic region leucine zipper (bZIP) transcription factors, which includes six members (α, β, γ, δ, ε and ζ) [16]. C/EBPα is required for proper control of adipogenesis, glucose metabolism, granulocytic differentiation, and lung development. Many studies demonstrate that C/EBPα indeed acts as a tumor suppressor in a number of tumor types [17]. In order to better understand the regulation of let-7a-1 in lung cancer cells, we cloned a 2.1 kb promoter fragment of let-7a-1 and made the preliminary analysis on its function in our previous work [18]. Our previous study shows that C/EBPα enhances the promoter activity of let-7a-1 [18]. Although a growing number of studies have demonstrated the regulation of specific miRNAs by C/EBPα [19–25], it is still unknown whether let-7 is the target of C/EBPα. In the present study, we investigate the influence of the C/EBPα on let-7a-1 regulation in lung cancer cells.

## Methods

### Bioinformatics

The DNA sequence of the 2.1 kb let-7a-1 promoter was analyzed with MatInspector 4.1 (http://www.genomatix.de). The MatInspector 4.1 software identifies putative transcription factor binding sites with weight matrices representing consensus recognition sequences for different transcription factors as defined in the MatInspector library [26]. Four CCAAT/enhancer binding protein sites upstream of the let-7a-1 start site were identified. They were termed CEBP1-4 in this work, respectively.

### Cells

Human lung cancer cell lines A549 and H1299, and human cervical cancer cell line Hela were obtained from Shanghai Institute of Biochemistry and Cell Biology, Chinese Academy of Science (Shanghai, China) and maintained in RPMI1640, F-12 K and DMEM (GIBCO, Thermo Fisher Scientific, Waltham, MA, USA) medium, respectively, supplemented with 10 % fetal bovine serum (FBS; GIBCO, Thermo Fisher Scientific, Waltham, MA, USA), in a humidified atmosphere with 5 % CO_2_ at 37 °C. Within 24 h of passage, all cells with more than 90 % confluence were used for transfection. X-tremeGENE HP (Roche, Mannheim, Germany) or Lipofectamin 2000 Transfection Agent (Thermo Fisher Scientific, Waltham, MA, USA) was used for plasmid or siRNA transfection, respectively, according to the manufacturer’s instructions.

### Plasmids

A 2.1-kb let-7a-1 promoter was cut from pGL3-2123 that was constructed in our previous work [18], and cloned into pGL4.10 [luc2] vector (Promega, Fitchburg, WI, USA) between Xho I and Hind III sites to form pGL4.10-2123. Four wild-type and mutant CEBP elements as well as CEBP consensus sequences were synthesized (Table 1). The double-stranded sequences were generated by annealing equal amounts of sense and antisense sequences at 95 °C for 10 min, and then cooling to room temperature. The double-stranded sequences with overhanging Xho I (tcgag) and Hind III (agctt) sites were inserted into the 5′ end of the luciferase gene in pGL4.23 [luc2/minP] vector to generate pGL4.23-CEBP1-4w/m, respectively. In the same way, the target sequence of let-7a (5′-UGAGGUAGUAGGUUGUAUAGUU-3′) was synthesized and inserted into sac I and Hind III sites of pMIR-Report luciferase reporter vector (Ambion, Thermo Fisher Scientific, Waltham, MA, USA) to generate pMIR-let7aT plasmid. All of the constructs were confirmed by DNA sequencing.

**Table 1:** Oligonucleotide sequences used for reporter gene assay

Name	Sequences (5′–3′)
CEBP consensus wild-type (CEBPcw)	Sense, *tcgag*TGCAGATTGCGCAATCTGCA*a*; anti-sense, *agctt*TGCAGATTGCGCAATCTGCA*c*
CEBP consensus mutant (CEBPcm)	Sense, *tcgag*TGCAGAGACTAGTCTCTGCA*a*; anti-sense, gcttTGCAGAGACTAGTCTCTGCAc
CEBP consensus wild-type (CEBP1w)	Sense, *tcgag*GAGGTTGTGAAACCC*a*; anti-sense, *agctt*GGGTTTCACAACCTC*c*
CEBP consensus mutant (CEBP1 m)	Sense, *tcgag*GAGGGAGTAAGCCCC*a*; anti-sense, *agctt*GGGTTTCACAACCTC*c*
CEBP2 wild-type (CEBP2w)	Sense, *tcgag*TGTGTTCTGTAAGCC*a*; anti-sense, *agctt*GGCTTACAGAACACA*c*
CEBP2 mutant (CEBP2 m)	Sense, tcgagTGTGGTCTATGCGCC*a*; anti-sense, *agctt*GGCGCATAGACCACA*c*
CEBP3 wild-type (CEBP3w)	Sense, *tcgag*TACGTTTTGTAATTT*a*; anti-sense, *agctt*AAATTACAAAACGTA*c*
CEBP3 mutant (CEBP3 m)	Sense, *tcgag*TACGGTTTATGCTTT*a*; anti-sense, *agctt*AAAGCATAAACCGTA*c*
CEBP4 wild-type (CEBP4w)	Sense, *tcgag*AAGATTCAGAAATCA*a*; anti-sense, *agctt*TGATTTCTGAATCTT*c*
CEBP4 mutant (CEBP4 m)	Sense, *tcgag*AAGAGTCAAAGCTCA*a*; anti-sense, *agctt*TGAGCTTTGACTCTT*c*

### Small interfering RNA (siRNA)

To knock down the expression of C/EBPα, Hela cells were transfected with si-h-C/EBPα-002 (RiboBio, Guangzhou, China) using Lipofectamine 2000 (Invitrogen, Thermo Fisher Scientific, Waltham, MA, USA). Non-targeting control siRNA (RiboBio, Guangzhou, China) and transfection reagents only (mock transfection) were transfected as negative controls. The siRNA was diluted to 50 nM in Opti-MEM I reduced-serum medium (Invitrogen, Thermo Fisher Scientific, Waltham, MA, USA). After transfection for 48 h, cells were collected to confirm the effect of C/EBPα knockdown at both mRNA expression levels and protein expression levels.

### Quantitative reverse-transcription polymerase chain reaction (qRT-PCR)

Total RNA was extracted from cells using Trizol reagent (Invitrogen, Thermo Fisher Scientific, Waltham, MA, USA) according to the manufacturer’s instructions. First strand cDNA was synthesized using a Revert Aid First Strand cDNA Synthesis Kit (Fermentas, Thermo Fisher Scientific, Waltham, MA, USA). The qRT-PCR amplification of pri-let-7a-1 was performed according to a previous method [5].

### Western blotting

Western blotting was performed to detect C/EBPα protein expression. Whole-cell lysates were prepared using lysis buffer [containing 50 mmol/L Tris-Cl (pH 8.0), 150 mmol/L NaCl, 0.1 % SDS, 1 %NP-40 and 100 mg/ml PMSF]. Protein concentration was determined using Bradford assay (Bio-Rad, Hercules, CA, USA). Extracted protein (30 μg) was processed in a 10 % SDS-PAGE gel and transferred to a polyvinylidene fluoride membrane. Protein levels of C/EBPα were measured by antibody raised against the C-terminus of human C/EBPα (sc-9314X, Santa Cruz Biotechnology, Dallas, TX, USA). Anti-β-Actin (Sigma, St. Louis, MO, USA) was used as an internal control. Immunoblots were detected using an enhanced chemiluminescence kit (Santa Cruz Biotechnology, Dallas, TX, USA) and visualized after exposure to X-ray film.

### Dual-luciferase reporter gene assay

Cells in 70–80 % confluence were co-transfected in 24-well plates. Each well contained 0.3 μg reporter gene plasmid, 0.02 μg of the internal control vector pGL4.74 [hRluc/TK] vector (Promega, Fitchburg, WI, USA), 1 μl transfection agent, and 0.2 μg expression vector or si-RNA. At 48 h after the completion of the transfection procedure, all the cells underwent dual-luciferase reporter assay, following the protocol recommended by the manufacturer’s manual. The cells were lysed using 1× reporter lysis buffer and harvested by manual scraping. Luminescence was detected using a Mithras LB 940 (Berthold Technologies, Oak Ridge, TN, USA). The firefly luciferase activity of the reporter gene plasmid was measure 1 (M1), and the renilla luciferase activity (internal control) of pGL4.74 [hRluc/TK] vector was measure 2 (M2). The relative luciferase activity was calculated as the ratio of M1/M2.

### Electrophoretic mobility shift assay (EMSA)

At 24 h before transfection, H1299 cells were inoculated in 6-well plate. When in 70–80 % confluence, each well was transfected by 0.2 μg pcDNA3.1(+)-C/EBPα. At 48 h after transfection, the cells were collected and prepared for nuclear extracts as described previously [27]. Double-stranded oligonucleotide probes were obtained by annealing sense and antisense sequences (Table 2). Probes were end-labeled with digoxin using T4 polynucleotide kinase (Invitrogen, Thermo Fisher Scientific, Waltham, MA, USA). EMSA was performed using the DIG Gel Shift Kit (Second Generation; Roche, Basel, Switzerland) following the manufacturer’s protocol. Antibodies against C/EBPα (sc-9314x; Santa Cruz Biotechnology, Dallas, TX, USA) was used for supershift assay.

**Table 2:** Oligonucleotide sequences used for electrophoretic mobility shift assay

Name	Sequences
CEBP consensus	5′-TGCAGATTGCGCAATCTGCA-3′
CEBP1 (−1726/−1712)	5′-gaaGAGGTTGTGAAACCCcct-3′
CEBP2 (−1684/−1670)	5′- tccTGTGTTCTGTAAGCCatc-3′
CEBP3 (−722/−708)	5′-tatTACGTTTTGTAATTTtaa-3′
CEBP4 (−163/−149)	5′-tacAAGATTCAGAAATCAccc-3′

### Chromatin immunoprecipitation (ChIP)

At 24 h before transfection, H1299 cells were inoculated in 10-cm dishes. When in 70–80 % confluence, each well was transfected by 2 μg pcDNA3.1(+)-C/EBPα or pcDNA3.1(+). At 48 h after transfection, the cells were collected and ChIP assay was performed using ChIP Assay Kit (Millippore, Billerica, MA, USA) following the manufacturer’s protocol. For each immunoprecipitation reaction, 10^6^ H1299 cells and 2 μg of goat anti-C/EBPα (sc-9314x; Santa Cruz Biotechnology, Dallas, TX, USA) or control IgG (Santa Cruz Biotechnology, Dallas, TX, USA) were used. The immunoprecipitated DNA was recovered and used as template for PCR using three pairs of primers complementary to the region flanking the C/EBPα motifs in let-7a-1 promoter (Table 3). The products were resolved on agarose gel, purified and sequenced. An aliquot of sheared chromatin DNA was subjected to PCR before immunoprecipitation and served as input control.

**Table 3:** Oligonucleotide sequences used for polymerase chain reaction primers in chromatin immunoprecipitation assay

Name	Sequences	Fragment lengths
CEBP1/2	Forward, 5′-ACAAACTTCACAGGTTGAGGGC-3′ reverse, 5′-TAATAAACCAGTGATAATGAGTGTCTTC-3′	184 bp
CEBP3	Forward, 5′-CAGCCGTCAGCATTATTTGT-3′ reverse, 5′-CGAAGATTATCTTTTAAGATAGGGA-3′	220 bp
CEBP4	Forward, 5′-GTAAAAGGTGGTGGTAAGAGGGT-3′ reverse, 5′-ACATGCATAATCTATGCTGTGGTT-3′	135 bp

### Statistical analysis

All data were analyzed using SPSS 17.0 software package (IBM, Armonk, NY, USA). All values were expressed as mean ± standard deviation from at least three independent experiments. Statistical significance was defined as P < 0.05.

## Results

### Positive correlation may exist between the expression levels of C/EBPα and pri-let-7a-1

**Fig. 1:**
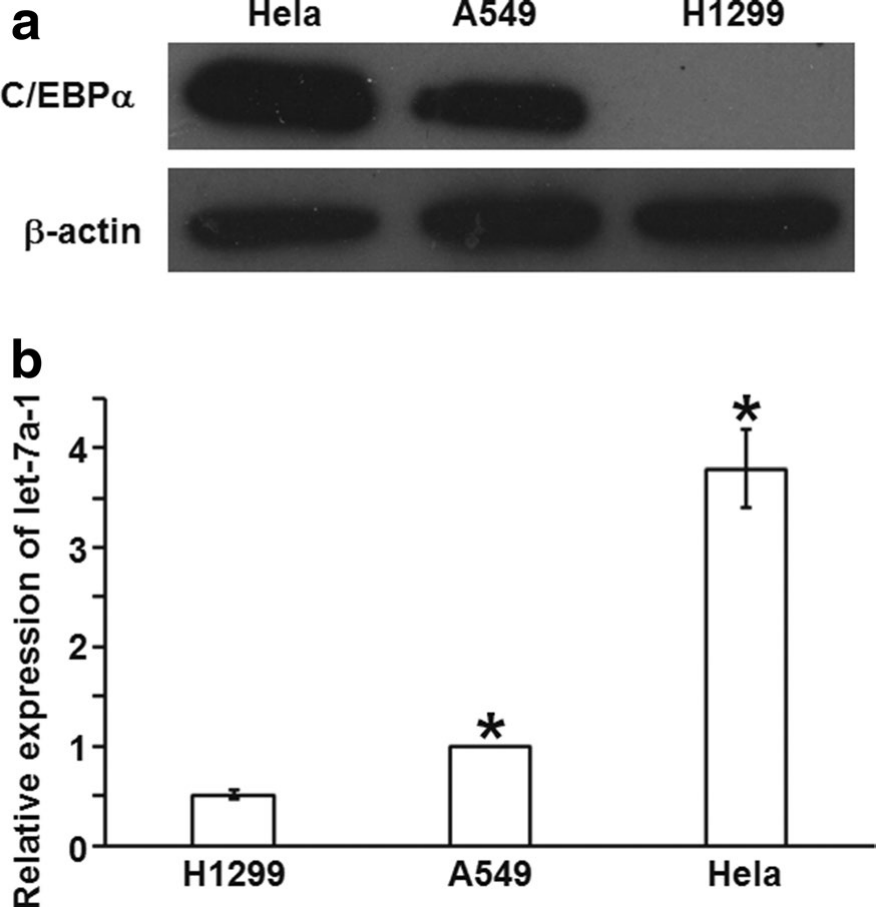
Expression of C/EBPα and let-7a-1. Detection of (**a**) C/EBPα protein (30 μg) expression and (**b**) let-7a-1 was performed by western blotting and quantitative real-time polymerase chain reaction, respectively, in Hela, A549 and H1299 cells. Data are represented as means ± standard deviations from three independent tests. *P < 0.05

To determine the expression levels of C/EBPα and pri-let-7a-1, western blotting and qRT-PCR were used, respectively. The data showed that C/EBPα and pri-let-7a-1 levels in A549 cells were significantly higher than that in H1299 cells, but were significantly lower than that in Hela cells (Fig. 1a, b). The result suggests that positive correlation may exist between the expression levels of C/EBPα and pri-let-7a-1.

### C/EBPα expression affects the promoter activity of let-7a-1 in a dose-dependent manner

**Fig. 2:**
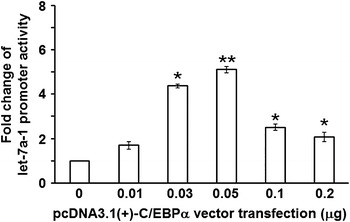
Effect of ectopic C/EBPα expression on promoter activity of let-7a-1 in H1299 cells. Data are represented as means ± standard deviations from three independent tests. *P < 0.05; **P < 0.005

To study how C/EBPα expression affects the promoter activity of let-7a-1, we performed reporter gene assay. The data showed that ectopic C/EBPα expression increased the promoter activity of let-7a-1 significantly in H1299 cells in a dose-dependent manner, reaching the maximal 5.11-fold at 0.05 μg vector transfection After that, the effect was weaken as the continued increase of transfection doses (Fig. 2). This result indicates that C/EBPα expression affects the promoter activity of let-7a-1 in a dose-dependent manner.

### C/EBP-α up-regulates the expression level of let-7a at transcription level

**Fig. 3:**
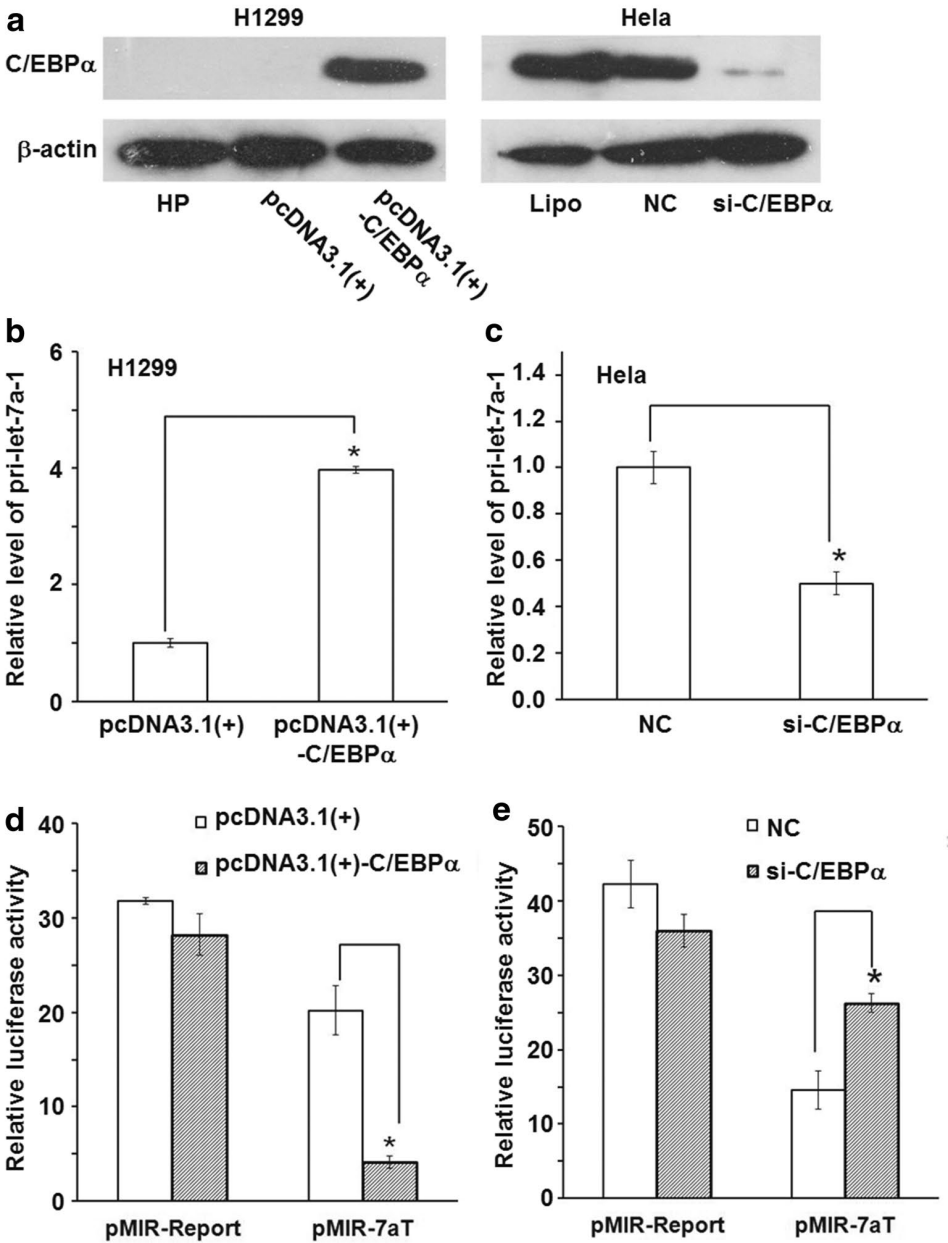
Effect of C/EBPα on the expression of let-7a-1. **a** Detection of ectopic C/EBPα overexpression in H1299 cells and knockdown in Hela cells by western blotting. For each sample, 30 μg total proteins were used. Detection of (**b**) induction of pri-let-7a-1 by ectopic C/EBPα expression in H1299 cells, and (**c**) reduction of pri-let-7a-1 by C/EBPα knockdown in Hela cells. Quantitative real-time polymerase chain reaction was performed to detect the level of pri-let-7a-1. Data are represented as means ± standard deviations from three independent tests. *P < 0.05. Detection of (**d**) let-7a induction by ectopic C/EBPα expression in H1299 cells, and (**e**) let-7a reduction by ectopic c/EBPα knockdown in Hela cells by Dual-luciferase reporter gene assay. Data are represented as means ± standard deviations from three independent tests. *P < 0.05

To further investigate whether the expression of let-7a-1 could be influenced by C/EBPα, H1299 cells were transfected with C/EBPα expression vector, while Hela cells were transfected with si-C/EBPα. First, the overexpression and knockdown of C/EBPα were confirmed by western blotting analysis (Fig. 3a). In addition, overexpression of C/EBPα increased the expression level of pri-let-7a-1 by 3.97-fold in H1299 cells (Fig. 3b), whereas knockdown of C/EBPα decreased the expression level of pri-let-7a-1 by about 50 % in Hela cells (Fig. 3c). Meanwhile, luciferase reporter assay showed that ectopic expression of C/EBPα significantly reduced luciferase activity in H1299 cells transfected with pMIR-7aT (Fig. 3d), while knockdown of C/EBPα significantly increased luciferase activity in Hela cells with si-C/EBPα transfection (Fig. 3e). These results suggest that C/EBP-α up-regulates the expression level of let-7a at transcription level.

### The let-7a-1 gene promoter contains potential binding sites for C/EBPα

**Fig. 4:**
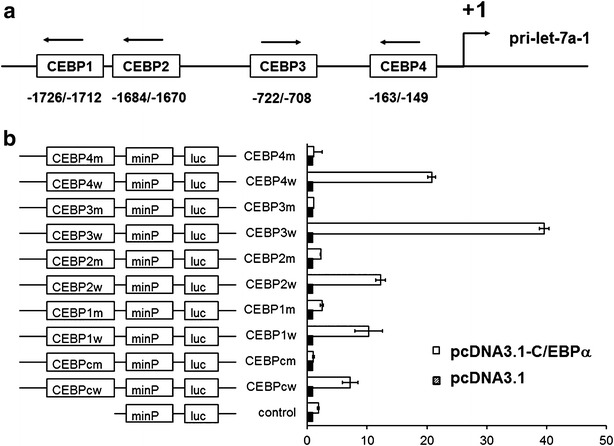
Four potential binding sites for C/EBPα in let-7a-1 gene promoter. **a** Schematic representation of four examined putative C/EBP binding sites in the promoter region of let-7a-1 predicted by MatInspector software. **b** Detection of the functions of four putative C/EBP elements by reporter gene assay. After wild-type and mutant sequences of CEBP1/2/3/4 elements as well as C/EBPα consensus sequence (CEBPcw/cm) were synthesized and inserted into reporter gene vector, the constructs were used to investigate the function and importance of these C/EBPα binding sites for let-7a-1 gene expression using dual-luciferase reporter gene assay. All of the plasmid constructs were transiently co-transfected into H1299 cells with pcDNA3.1(+)-C/EBPα, using pcDNA3.1(+) as control

According to MatInspector with Matrix Similarity 0.85 and Matrix Family Library, there are four C/EBP potential binding sites within 2.1 kb of human let-7a-1 promoter upstream of the transcription start site. These four C/EBP potential binding sites were termed as CEBP1/2/3/4, where CEBP1/2 sites were in tandem within a 57-bp sequence (−1726/−1670) (Fig. 4a). After wild-type and mutant sequences of CEBP1/2/3/4 elements as well as C/EBPα consensus sequence (CEBPcw/cm) were synthesized and inserted into reporter gene vector, the constructs were used to investigate the function and importance of these C/EBPα binding sites for let-7a-1 gene expression using dual-luciferase reporter gene assay. All of the plasmid constructs were transiently co-transfected into H1299 cells with pcDNA3.1(+)-C/EBPα, using pcDNA3.1(+) as control. Compared to co-transfection with pcDNA3.1(+), all wild-type C/EBP elements co-transfected with pcDNA3.1(+)-C/EBPα presented significantly increased luciferase activity. Moreover, the increase of C/EBP elements within let-7a-1 was higher than C/EBP consensus. However, mutant C/EBP elements displayed insignificant change in luciferase activity in response to the ectopic expression of C/EBPα (Fig. 4b). These results indicate that let-7a-1 gene promoter contains potential binding sites for C/EBPα.

### C/EBPα can bind let-7a-1 promoter as demonstrated by EMSA

**Fig. 5:**
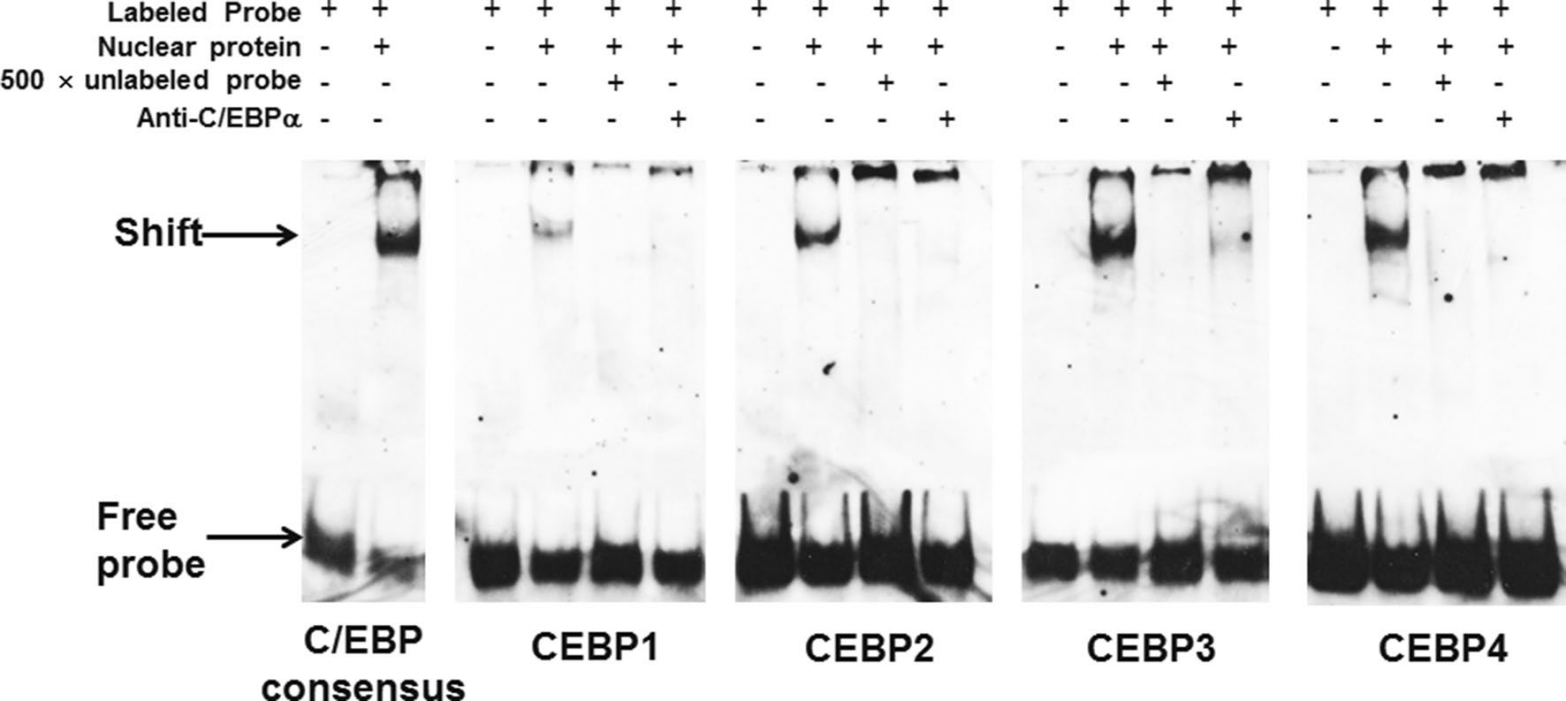
Binding of the four C/EBP elements with C/EBPα. EMSA experiments were performed with nuclear extracts from H1299 cells with ectopic C/EBPα expression, using C/EBP consensus sequence as positive control

To determine whether these putative C/EBP elements are capable of binding transcription factor C/EBPα in vitro, we performed a series of EMSA experiments with nuclear extracts from H1299 cells with ectopic C/EBPα expression, using C/EBP consensus sequence as positive control. All of the four CEBP probes and C/EBP consensus sequence showed transcription factor binding activity. Furthermore, these complexes were blocked specifically by anti-C/EBPα antibody and were effectively competed by cold competitor oligonucleotides (Fig. 5). The result suggests that C/EBPα can bind let-7a-1 promoter.

### The let-7a-1 promoter is a direct transcriptional target of C/EBPα as determined by ChIP assay

**Fig. 6:**
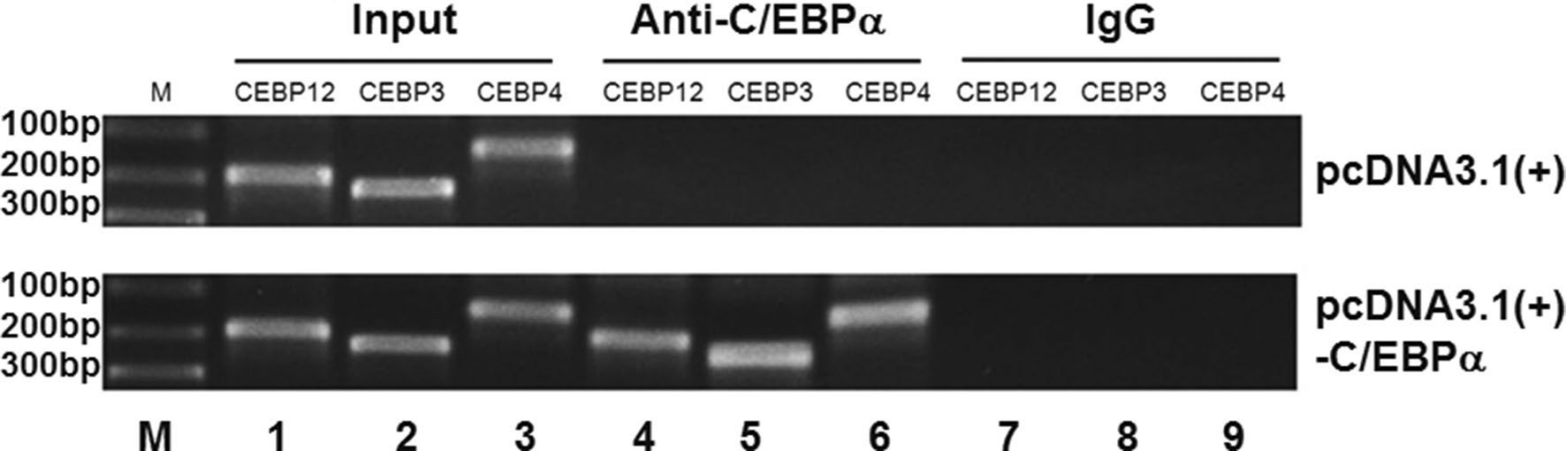
Targeting of let-7a-1 promoter by C/EBPα. ChIP assay was performed on H1299 cells transfeted with pcDNA3.1(+) or pcDNA3.1(+)-C/EBPα. C/EBPα proteins were immunoprecipitated from DNA–protein cross-linked cell lysates. After the removal of all proteins, the recovered DNA was precipitated by ethanol and amplified by PCR using primers specific for let-7a-1 promoter

In order to further confirm that C/EBPα indeed binds to let-7a-1 promoter, we performed endogenous ChIP. First, C/EBPα proteins were immunoprecipitated from DNA–protein cross-linked cell lysates. After the removal of all proteins, the recovered DNA was precipitated by ethanol and amplified by PCR using primers specific for let-7a-1 promoter. In contrast to H1299 cells transfected with pcDNA3.1(+), H1299 cells transfected with pcDNA3.1(+)-C/EBPα showed that all of four C/EBP sites within let-7a-1 promoter were indeed associated with C/EBPα immunoprecipitates (Fig. 6, lanes 4–6), whereas a IgG negative control failed to precipitate any let-7a-1 promoter DNA (Fig. 6, lanes 7–9). The result indicates that let-7a-1 promoter is a direct transcriptional target of C/EBPα.

## Discussion

miRNAs are a group of gene regulators that play important roles in biologic processes such as cell proliferation, differentiation, and apoptosis. The dysregulation of miRNAs is associated with the development of cancers, including lung cancer [5, 28, 29]. Many evidences have indicated that miRNA genes are transcribed by RNA polymerase II, and only a few miRNA promoters in *H. sapiens* have been identified so far. As class II genes, expressions of many miRNAs are also regulated by enhancers or hormones [30–32]. As a result, one of the reasons for the dysregulation of miRNAs in tumor tissues or cells may be the dysregulation at transcription level.

C/EBPα is a basic leucine zipper transcription factor that is expressed in many tissues, and plays an important role in regulating the balance between cell proliferation and cell differentiation [17, 33]. Loss of C/EBPα functions has been linked to leukemogenesis, lung cancer, liver cancer, etc., suggesting an important role for C/EBPα as a tumor suppressor [34, 35]. A growing number of studies have demonstrated the regulation of specific miRNAs by C/EBPα. Fazi et al. first identify miR-223 as a direct target of C/EBPα, and C/EBPα-induced up-regulation of miR-223 leads to granulopoiesis [19]. Zeng et al. report that C/EBPα directly interacts with miR-122 (hepatocyte-specific miRNA) promoter and transactivates it [20]. Moreover, recent studies show that several tumor suppressor miRNAs are directly regulated by C/EBPα, such as miR-223, miR-34a and miR-30C, and that transactivation of all miRNAs is inhibited along with the down-regulated expression of C/EBPα [21–23]. In A549 and H1299 lung cancer cells, ectopic C/EBPα expression increases the expression of miR-1 by 6.1 and 4.92-fold, respectively [24]. miR-661 is another C/EBPα target [25].

Our previous study shows that C/EBPα enhances the promoter activity of let-7a-1 gene in lung cancer A549 cells [18]. In the present study, we identify that let-7a-1 gene is another new target of C/EBPα. We first examined the association between pri-let-7a-1 and C/EBPα expression. The expression levels of C/EBPα and pri-let-7a-1 are found to be down-regulated in several lung cancer cell lines [5, 36], whereas Hela cells have high level of let-7 expression. Therefore, we selected lung cancer cell lines A549 and H1299, as well as and Hela cells to detect expression levels of the two by qRT-PCR and western blotting. The results show that pri-let-7a-1 level is positive correlated with C/EBPα expression. Furthermore, the ectopic expression of C/EBPα increases pri-let-7a-1 level in H1299 cells, while RNA interference for C/EBPα decreases pri-let-7a-1 level in Hela cells. The results demonstrate that C/EBPα transactivates the expression of let-7a-1 gene. Based on the fact that let-7a-1 is the most abundant specie of let-7a subfamily, reporter gene assay is performed to observe the effect of C/EBPα on mature-let-7a expression. Dual-luciferase detection shows that ectopic expression of C/EBPα increases mature-let-7a level in H1299 cells, while RNA interference for C/EBPα decreases mature-let-7a level in Hela cells. Moreover, reporter gene assay performed in H1299 cells has displayed that ectopic expression of C/EBPα increases promoter activity of let-7a-1, being in accordance with our previous result [18]. Therefore, the transcription factor C/EBPα can up-regulate the expression of let-7a-1 gene at transcription level.

Bioinformatics assay has identified four putative C/EBP elements within let-7a-1 promoter. We infer that at least one of them may mediate the transactivation effect of C/EBPα. To confirm this, luciferase activity assay was performed in H1299 cells to analyze the function of the four putative CEBP elements. The results showedthat all of the four elements respond to ectopic expression of C/EBPα, and their luciferase activities are higher than that of C/EBP consensus sequence used as positive control. Among them, CEBP3 element has displayed the highest activity. Furthermore, in vitro binding assay shows that all of the four C/EBP elements and C/EBP consensus sequence have binding capacity with nuclear protein extracted from H1299 cells with ectopic C/EBPα expression. In addition, these binding complexes can be blocked by anti-C/EBPα antibody specifically. At last, ChIP assay demonstrates the direct interaction between C/EBPα and the four C/EBP elements. In summary, the present study identifies tumor suppressor miRNA let-7a-1 gene as a new direct target of transcription factor C/EBPα. Our results conclude that decrease or absence of tumor suppressor C/EBPα contributes to the transcriptional inhibition of let-7a-1.
